# Editorial: Metal transport in plants, volume II

**DOI:** 10.3389/fpls.2024.1395567

**Published:** 2024-04-03

**Authors:** Stephan Clemens, Seckin Eroglu, Louis Grillet, Tomoko Nozoye

**Affiliations:** ^1^Department of Plant Physiology, University of Bayreuth, Bayreuth, Germany; ^2^Department of Biological Sciences, Middle East Technical University, Ankara, Türkiye; ^3^Department of Agricultural Chemistry, National Taiwan University, Taipei, Taiwan; ^4^Center for Liberal Arts, Meiji Gakuin University, Tokyo, Japan; ^5^Graduate School of Agricultural and Life Sciences, The University of Tokyo, Tokyo, Japan

**Keywords:** metal, transport, translocation, plant, essential nutrient elements, toxic metal

All biological compounds are made of elements. Human beings belong to the heterotrophs and cannot produce energy. On Earth, plants are principal autotrophs that can produce food by photosynthesis to supply energy for organisms. Ultimately, the elements plants take up and accumulate from the environment become part of our bodies’ composition. Therefore, an understanding of elemental transport in plants is essential for both solving agricultural problems and improving human health. Plants have to acquire 17 different essential elements (nitrogen, phosphorus, potassium, oxygen, hydrogen, carbon, calcium, magnesium, sulfur, iron, manganese, boron, zinc, molybdenum, copper, chlorine, and nickel) mainly from the rhizosphere by their roots to complete their life cycle. Both shortage and excess of these elements lead to impaired growth, which often translates into yield loss in agriculture. Plants have developed sophisticated and tightly regulated mechanisms for the uptake and translocation of essential elements. On the other hand, plants also take up harmful elements such as cadmium and arsenic non-specifically which may interfere with essential nutrients’ uptake. In this Research Topic, we have aimed to cover essential nutrient homeostasis in plants; including, but not limited to their transport, translocation, and interaction with each other. In the Research Topic Volume I, we succeeded in collecting six original research papers, one review, one perspective, and one mini-review, which discuss not only the essential but also the toxic metals under various physiological conditions. Following the publication of the Research Topic on *Metal transport in plants, Volume I*, we decided to edit a second volume of our Research Topic, which indicates the importance and significant progress in this research field.

The Research Topic Volume II includes five original research papers, one review, and one mini-review. Bakirbas and Walker found *CAN OF SPINACH* (*COS*), a novel long non-coding RNA (lncRNA) involved in iron (Fe) homeostasis. They performed a comparative RNAseq analysis of Fe sufficient, deficient, and resupplied (24 hours Fe resupply after Fe deficiency treatment) Arabidopsis shoots, and focused on a particular Fe-regulated transcript, named *COS*. Bakirbas and Walker found that reads matching this locus originated from previously unannotated transcripts on the reverse strand which did not contain cDNA and were not produced from the strand predicted to encode proteins. The *cos* mutant has low Fe levels in the leaves, although it has higher chlorophyll levels compared to wild type (WT). In addition, the *cos* mutant accumulated singlet oxygen during Fe deficiency, which caused negative effects on photosynthesis. It was suggested that *COS* lncRNA negatively regulates several Fe homeostasis genes.

Liu et al. have revealed that the maize Lazarus (LAZ1) 1-4 transporter is involved in zinc (Zn) homeostasis. Expression of *ZmLAZ1-4* significantly inhibited the growth of Zn-sensitive yeast mutants, suggesting that ZmLAZ1-4 could transport Zn into the cells. Overexpression of *ZmLAZ1-4* increased Zn concentrations in seedlings without causing growth defects both in Arabidopsis and maize. In maize protoplast and onion epidermal cells, ZmLAZ1-4 localized to plasma and vacuolar membrane, as well as the chloroplast. Liu et al. also confirmed that *ZmLAZ1-4* was negatively regulated by transcription factor maize BRASSINOSTEROID INSENSITIVE 1- ethyl methanesulfonate- SUPPRESSOR 1 (BES1)/BRASSUBAZOLE RESISTANT 1 (BZR1) (ZmBEZ1/BZR1-11) using co-expression analysis, yeast one-hybrid assay and dual-luciferase assay *in vivo*. It was suggested that ZmLAZ1-4 is a novel Zn transporter that modulates Zn homeostasis in maize.

Yang et al. analyzed the function of Ethylene Response Factor109 (ERF109) and found that cellular Fe homeostasis and immunity regulatory networks are connected via ERF109 in leaves. *erf109* mutants contain higher Fe in the leaves, while the maximum quantum yield of photosystem II, the quantum yield of PSI, and SPAD values (a read-out for chlorophyll content) were lower compared to WT. RNA seq analysis of the leaves showed that *erf109* mutants showed symptoms of Fe deficiency even under Fe-sufficient conditions since the transcriptome of *erf109* under Fe-sufficient conditions was similar to that of Fe-deficient WT. In addition, the regulation of Fe-responsive immunity-associated genes or genes encoding photosystem subunits and chlorophyll biosynthesis enzymes that are part of the Fe deficiency response was abolished in *erf109* mutants compared to WT. Yang et al. hypothesized that compromised expression of *ERF109* perturbs Fe homeostasis at an early stage of the signaling cascade by repressing the Fe acquisition machinery.

Muro et al. revealed that Casparian strips are important for preventing apoplastic diffusion of boron (B) into the root steles under excess B supply by analyzing Arabidopsis mutants with a defective Casparian strip structure, namely *sgn3* and *sgn4*. *sgn3* or *sgn4* are defective in the receptor-like kinase SCHENGEN3 (SGN3) or NADPH oxidase SGN4/respiratory burst oxidase homolog F (RBOHF), respectively. SGN3 and SGN4 are both required for the structure of Casparian strips. *sgn3* and *sgn4* mutants showed growth defects and increased B accumulation in shoots as well as faster translocation of B into the root stele and shoots compared to WT when excess B was supplied. These results suggested that Casparian strips contribute to tolerance to excess B by preventing B translocation under high-B conditions.

Uraguchi et al. have succeeded in enhancing the tolerance of Arabidopsis to mercury (Hg) by introducing a bacterial mercury transporter *MerC* under control of mesophyll-specific RubisCO-related promoter *pRBCS1A*. The transgenic plants are expected to be good candidates for phytoremediation of Hg-contaminated soil. For efficient phytoremediation, target metals need to be accumulated in the shoots, ideally without impairing growth. By introducing *MerC* fused to *AtVAM3*, a tonoplast-resident syntaxin, under the control of *pRBCS1A*, Arabidopsis plants became more tolerant to Hg compared to WT. The fresh weight of these plants increased under Hg exposure conditions compared to WT. However, Hg concentrations were not changed. Uraguchi et al. suggested that increasing Hg uptake by roots could boost plant Hg accumulation.

In the review, Park and Shin summarize and discuss recent discoveries of various membrane proteins involved in calcium (Ca) influx or efflux and their essential role in plant responses to abiotic and biotic stress. Tabata summarized in a mini-review recent advances in understanding how IRON MAN/FE-UPTAKE-INDUCING PEPTIDES (IMA/FEP) function in the intracellular signaling of the Fe-deficiency response and systemic Fe signaling to regulate Fe acquisition.

The present Research Topic illustrates the important role several elements play in the regulation of abiotic and biotic stress responses. Especially, our understanding of photosynthesis and root-to-shoot communication is rapidly evolving thanks to cutting-edge genomic tools. Plants cannot move, and take up elements mainly through their roots and translocate them to shoots. In addition, plants cannot escape from their fluctuating environment and need to adapt. Understanding metal transport systems will continue to yield important insight into how we should adapt to a constantly and rapidly changing Earth.

## Author contributions

SC: Writing – review & editing. SE: Writing – review & editing. LG: Writing – review & editing. TN: Writing – original draft.

